# Effectiveness of a COVID-19 Testing Outreach Intervention for Latinx Communities: A Cluster Randomized Trial

**DOI:** 10.1001/jamanetworkopen.2022.16796

**Published:** 2022-06-16

**Authors:** David S. DeGarmo, Stephanie De Anda, Camille C. Cioffi, Hannah F. Tavalire, Jacob A. Searcy, Elizabeth L. Budd, Ellen Hawley McWhirter, Anne Marie Mauricio, Sven Halvorson, Emily A. Beck, Llewellyn Fernandes, Mark C. Currey, Jorge Ramírez García, William A. Cresko, Leslie D. Leve

**Affiliations:** 1 Prevention Science Institute, University of Oregon, Eugene; 2 Department of Counseling Psychology and Human Services, University of Oregon, Eugene; 3 Department of Special Education and Clinical Sciences, University of Oregon, Eugene; 4 Presidential Initiative in Data Science, University of Oregon, Eugene; 5 Institute of Ecology and Evolution, University of Oregon, Eugene; 6 Oregon Research Institute, Eugene

## Abstract

**Importance:**

Latinx individuals have been disproportionately affected during the COVID-19 pandemic caused by the spread of SARS-CoV-2. It is imperative to evaluate newly developed preventive interventions to assess their effect on COVID-19 health disparities.

**Objective:**

To examine the effectiveness of a culturally tailored outreach intervention designed to increase SARS-CoV-2 testing rates among Latinx populations.

**Design, Setting, and Participants:**

In this cluster randomized trial performed from February 1 to August 31, 2021, in community settings in 9 Oregon counties, 38 sites were randomized a priori (19 to the community health promoters intervention and 19 to outreach as usual wait-listed controls). Thirty-three sites were activated. A total of 394 SARS-CoV-2 testing events were held and 1851 diagnostic samples collected, of which 919 were from Latinx persons.

**Interventions:**

A culturally informed outreach program was developed that made use of *promotores de salud* (community health promoters) to increase Latinx SARS-CoV-2 testing. Strategies addressed barriers by disseminating information on testing events in English and Spanish, mitigating misinformation, and increasing trust.

**Main Outcomes and Measures:**

The primary outcomes were the count of sample tests from Latinx persons and the sampled proportion of the Latinx populace. Site-level covariates included census tract Latinx populace, nativity (number of US-born individuals per 100 population), median age, and income inequality. Time-varying covariates included number of new weekly SARS-CoV-2–positive cases and percentage of vaccine coverage at the county level.

**Results:**

A total of 15 clusters (sites) were randomized to the control group and 18 to the community health promoters group. A total of 1851 test samples were collected, of which 995 (53.8%) were from female participants and 919 (49.6%) were from Latinx individuals. The intervention tested 3.84 (95% CI, 2.47-5.97) times more Latinx individuals per event than controls (incident rate ratio, 0.79; 95% CI, 0.46-1.34; Cohen *d* = 0.74; *P* < .001). The intervention was associated with a 0.28 increase in the proportion of Latinx populace being tested compared with control sites for the dependent variable scaled as the proportion of the Latinx populace ×100, or a 0.003 proportion of the raw populace count. The use of a standardized scaling of the proportion of Latinx individuals showed that the relative percentage increase was 0.53 (95% CI, 0.21-0.86) in the intervention sites compared with controls, representing a medium effect size.

**Conclusions and Relevance:**

To our knowledge, this was the first randomized evaluation of an outreach intervention designed to increase SARS-CoV-2 testing among Latinx populations. Findings could be used to implement strategies to reduce other health disparities experienced by these groups.

**Trial Registration:**

ClinicalTrials.gov Identifier: NCT04793464

## Key Points

**Question:**

Can a culturally informed community-based outreach intervention increase Latinx participation at SARS-CoV-2 testing events?

**Findings:**

In this cluster randomized trial of 33 SARS-CoV-2 testing sites, the community health promoters intervention was associated with 3.84 times more Latinx individuals tested per event than control sites, and the intervention was associated with testing a greater proportion of the Latinx populace per event.

**Meaning:**

The reduction of health disparities experienced by individuals identifying as Latinx during the COVID-19 pandemic may be supported by culturally informed outreach strategies.

## Teaser

This cluster randomized trial assesses the effect of a culturally tailored outreach program on SARS-CoV-2 testing rates among Latinx populations in Oregon.

## Introduction

Data from 36 countries have indicated that population testing frequencies for SARS-CoV-2 (the virus that causes COVID-19) below 15% are associated with exponentially increasing rates of mortality.^1^ Data from Our World in Data of 27 countries indicate that early testing capacity defined as tests per cases is associated with lower COVID-19–related mortality rates.^2^ In the first quarter of 2020, the National Institutes of Health (NIH) considered SARS-CoV-2 testing so important that the Rapid Acceleration of Diagnostics (RADx) initiative was launched to develop rapid and accurate testing and increase availability nationwide.^3^ As part of that mission, the RADx Underserved Populations focused on solutions to stop the spread of COVID-19 among racially and ethnically diverse populations who were disproportionately affected.^3^

In 2020, Latinx individuals were consistently overrepresented among cases,^4^ experiencing 3 times higher COVID-19–related mortality relative to non-Latinx White counterparts.^5^ Currently, Latinx individuals represent 19% of the US population but still comprise 28% of COVID-19 cases nationally.^6^ One year into the pandemic, Latinx Oregon residents did not fare better. Making up 13% of the state population, Latinx individuals represented 34% of COVID-19 cases and 25% of COVID-19 deaths.^7^ Moreover, nationally representative data indicate that Latinx individuals were underrepresented at testing sites across the US in early 2020,^8^ and for every 1% increase in underrepresentation of Latinx persons based on testing site zip codes, a state’s mortality rate was 1.04 percentage points more overrepresentative compared with non-Latinx individuals.

The purpose of this report is to evaluate SARS-CoV-2 testing rates from an effectiveness trial called *Oregon Saludable: Juntos Podemos* (Healthy Oregon: Together We Can) (OSJP). The OSJP *Promotores de Salud* (community health promoters, or *promotores*) intervention includes a culturally tailored outreach designed to increase testing among the Latinx population. *Promotores* models have been successful in addressing other community mental and physical health outcomes (eg, hypertension, depression, and chronic disease)^9,10,11^ but have not been evaluated for SARS-CoV-2 testing. We note that ethnic and cultural labels change over time, and there is often disagreement among group members.^12^ Although this study’s self-identified ethnicity included established NIH terms and country of origin, we use Latinx throughout instead of Hispanic or Latino, considered by some to be gender neutral and more inclusive for this diverse ethnic group.^13^

Underrepresentation of Latinx communities in testing is influenced by factors such as lack of understanding of available resources, language barriers, and access challenges.^14,15^ For immigrant, migrant, and Indigenous Latinx individuals, lack of trust in institutions and misinformation also reduce the likelihood of testing.^16^ Reducing testing barriers and overcoming health disparities for Latinx communities require culturally responsive interventions, including not requiring health insurance, physician orders, identification, or fees and offering walk-up service.^15,17^ Although several programs to reduce barriers to SARS-CoV-2 testing for Latinx communities exist,^14,15,17,18^ to our knowledge, this is the first randomized trial to evaluate strategies designed to accelerate SARS-CoV-2 testing among Latinx populations. On the basis of the community-based participatory intervention development and literature reviewed, we hypothesized that intervention (*promotores*) sites would be associated with higher numbers of Latinx individuals tested over time per site relative to county and community outreach as usual (OAU) and a higher proportion of the Latinx populace tested compared with control sites.

## Methods

### Study Design

This cluster randomized trial used wait-listing to enroll participant sites.^19^ Testing participants and testing staff were blind to the intervention condition, but community-based organizations (CBOs) and county health agencies were not. Participants provided a written waiver for the use of deidentified count totals for each testing event. All consent procedures and protocols were reviewed and approved by the Committee for Protection of Human Subjects and the University of Oregon Institutional Review Board. The trial protocol is available in Supplement 1. This study followed the Consolidated Standards of Reporting Trials (CONSORT) reporting guideline extension for cluster randomized trials.

Several steps were taken for site-level randomization. First, a facilities-location-problem approach^20^ was used to optimize 38 site locations in 9 Oregon counties with geomapping Latinx population concentrations to determine potential locations for testing events. Second, we focused the community engagement collaboration on instrumental aspects of testing, such as site access, optimizing visibility of events, and ensuring overall perceived safety (eg, real or perceived antagonism by community members opposed to testing, virus transmission during events, or US Immigration and Customs Enforcement authorities showing up). Given less than 10 participating counties with up to 6 possible sites, using a random-number generator, we randomized within county to minimize threats to internal validity.^19^ Sites were randomized to either the intervention group or the OAU wait-listed control group.

### *Promotores de Salud* Outreach

The intervention used a community-based participatory approach that relied on partnerships with ongoing knowledge exchange among researchers, stakeholders, and the community toward development of a culturally responsive intervention.^21^ *Promotores* were bilingual (Spanish and English) and bicultural community members (N = 19) recruited through close partnerships with and hired by CBOs (eg, regional farm worker advocacy center, nonprofit organization providing integrated social services, and advocacy groups for rural underrepresented populations). A subset of the *promotores* consented and provided demographic information (n = 16). Of those, 7 (44%) had completed high school or General Educational Development, and 5 (31%) had some college or an associate’s degree. A total of 8 (50%) had lived in the US their entire life, and 7 (44%) had lived in the US at least half of their life.

*Promotores* were trained to conduct outreach that highlighted common Latinx cultural values (eg, collective welfare); to disseminate information on testing events in Spanish, reasons to get tested, and COVID-19–related resources; to mitigate misinformation; and to increase trust. Strategies were tailored to local communities, including promoting testing via texting, in-person promotion at locations frequented by Latinx members (ie, specialty grocery stores, Spanish-language church services, schools, and workplaces), and advertising in print media and Latinx radio stations. All social media posts, flyers, and print outreach materials were prepared in Spanish and English. Regular meetings were held with Latinx community partners, the Oregon Health Authority, county health agencies, community and scientific advisory boards, CBOs, and *promotores* to (1) share up-to-date information and resources about the state’s pandemic mitigation strategies, (2) plan testing event locations vis-à-vis other regional COVID-19 mitigation events, and (3) problem solve and continuously share outreach strategies twice weekly with regional CBOs. Interpreters for Mam, an Indigenous Mayan language used in Oregon, were onsite at some locations.

### Outreach as Usual

Staff sample collection and testing procedures were the same across conditions. Both conditions held recurring testing events that ran for 3 to 4 hours every 2 weeks at the same time and location when possible. The OAU condition included community advertising by the study team using flyers in Spanish and English that were regularly distributed to a statewide listserv, posted on Facebook, and shared by community partners.

### Sample Collection and Testing

Anterior nares swabs were self-collected under guidance of trained study staff, placed in buffering agent, and transported to the laboratory. SARS-CoV-2 diagnostic testing was conducted at the University of Oregon’s Clinical Laboratory Improvement Amendments–certified laboratory using the US Food and Drug Administration emergency use–authorized TaqPath quantitative polymerase chain reaction assay (Thermo Fisher Scientific) and analyzed using the COVID-19 Interpretive Software (Applied Biosystems) with 3 outcomes: SARS-CoV-2 positive, SARS-CoV-2 not detected, or uncertain. Test results were returned to patients by secure email, accessed through a secure online portal, or distributed by US postal mail. Communications were available in Spanish, English, and Mam with links to posttest COVID-19 resources.

### Sample

**Figure 1.**
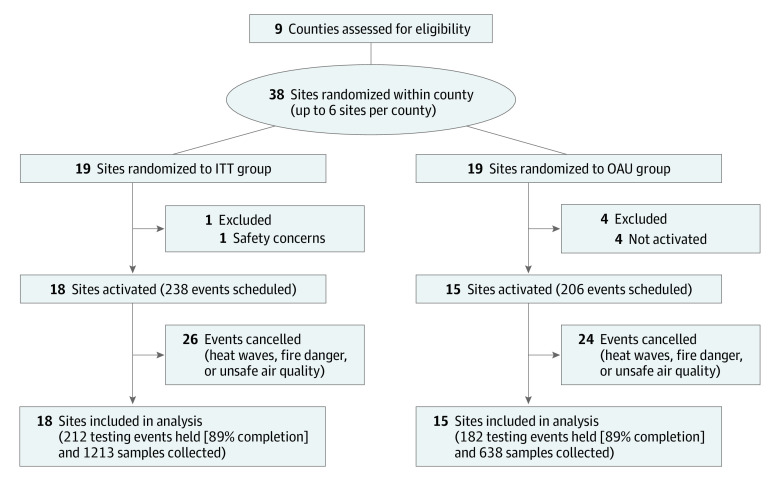
Study Flow Diagram of Enrollment, Randomization Sites, and Follow-up of Testing Samples for Cluster Randomized Trial ITT indicates intention-to-treat; OAU, outreach as usual.

Thirty-eight testing sites were randomized (19 *Promotores de Salud* sites and 19 OAU sites). Data were collected from February 1 to August 31, 2021, at which time wait-listed control sites received the intervention. A total of 444 testing events were scheduled. Fifty events (11%) were canceled because of competing events held at a testing site or extreme weather conditions, including heat waves, high winds, fire danger, and unsafe air quality because of smoke, for a total analysis sample of 394 events. Of those, the final analysis file included 212 *Promotores de Salud* sites and 182 OAU events. Persons 3 years or older were eligible (Figure 1).^22,23^

### Measures

The primary outcomes of the registered trial were number of completed SARS-CoV-2 diagnostic samples collected from Latinx individuals at each testing event and proportion of the Latinx populace tested. Self-identified individual data on biological sex, gender, and racial and ethnic characteristics were collected via questionnaire during testing registration. Race and ethnicity data were collected with superordinate and subordinate classifications. Superordinate categories included Asian, Black or African American, Hispanic and Latino/a/x, Indigenous American Indian or Alaska Native, Middle Eastern or North African, and Native Hawaiian or Pacific Islander. The proportion of the Latinx populace that was tested was computed as the number of samples collected from Latinx individuals divided by the number of Latinx individuals per site census tract.

### Census Tract Covariates

Five site-level census tract variables were matched to the testing site address with geocoded *x*-*y* coordinates for latitude and longitude. The census tract data^24^ were matched to site addresses using Federal Information Processing Standards geocoding. Covariates included estimated count of Latinx population (number of Latinx individuals per 100 population), nativity (number of US-born individuals per 100 population), median age, and income inequality measured with the Gini index, ranging from 0 (0%) to 1 (100%), with 0 representing perfect equality and 1 representing perfect inequality.

### Weekly County SARS-CoV-2 Covariates

Two time-varying covariates potentially affecting testing rates were extracted from the Oregon Health Authority database.^25,26^ The SARS-CoV-2 transmission rate was measured by the total number of new weekly cases per county, and the total county population vaccination coverage was measured as the percentage of completed series of vaccinations. The weekly number of new cases was log transformed to help meet the assumption of homogeneity of variance among variables. Both the weekly cases and vaccine coverage were lagged by 1 week and then matched to the week of the testing event to meet time-ordered causal assumptions. In addition, we controlled for whether a site offered vaccines at an event. We did not offer vaccines; however, we partnered with county organizations that did so at 4% of events.

### Statistical Analysis

Effectiveness hypotheses were tested with multilevel or mixed-model regressions. The count outcome (number of Latinx sample tests) was specified as a negative binomial generalized linear model to address both the repeated event data per site and overdispersed count data as log of the expected number of Latinx samples collected as follows:

Log[E(Latinx Sample Count)] = γ_00_ + γ_01_(Intervention Contrast) + γ_02_(Latinx Populace) + γ_03_(Vaccine Offered) + γ_04_(Native Born) + γ_05_(Income Inequality) + γ_06_(Median Age) + γ_10_(Lagged Log of New Cases) + γ_20_(Lagged Vaccination Coverage) + *u*_0_ + *u*_1_ + *r*,

where log sample count is regressed on the level 2 intervention effect γ_01_, site census covariates γ_02_ to γ_05_, level 1 time-varying weekly cases from prior week γ_10_, and county vaccine coverage from prior week γ_20_ plus residuals for predicted model (*u*_0_), level 1 (*u*_1_), and level 2 (*r*). The proportion of the Latinx populace tested was estimated as a linear mixed model using the same equation above. Models were estimated in the R GLMMadaptive package^27^ and tested with a 2-tailed α level and *P* < .05.

## Results

The number of testing events, sample tests collected, and samples from Latinx persons are given in Table 1. Table 1 presents repeated event time-varying means for test samples and weekly county COVID-19 data. Site-level characteristics are also presented. No significant differences were found among county cases, vaccine coverage, vaccine events, any of the site-level census characteristics, or number of repeated events held. Moreover, no significant differences were found in cancellations by condition (χ^2^_1_ = 0.05, *P* = .81). Site types included 15 schools (45.5%), 7 places of worship (21.2%), 4 school district offices (12.1%), 4 workplaces (12.1%), 1 public park (3.0%), 1 community center (3.0%), and 1 college (3.0%). A mean (SD) of 11.78 (3.17) repeated testing events were held for the intervention sites during the study period, and 12.13 (2.13) repeated events were held for controls. In total, 1851 participants were sampled; 1213 (66%) were from *Promotores de Salud* sites and 638 (34%) from OAU sites, for a mean (SD) of 4.70 (8.02) tests per event (5.72 [9.77] for the intervention sites and 3.51 [5.07] for the control sites). A total of 919 samples (49.6%) were from Latinx individuals, with a mean (SD) of 3.03 (3.44) samples from Latinx individuals in the intervention sites and 1.52 (2.48) in the control sites. Overall, 85 samples (4.6%) were from individuals who identified as American Indian or Alaska Native; 746 (40.3%), as White; and 57 (3.1%), as more than 1 race or ethnicity. A total of 913 samples (49.3%) were from individuals with unknown race and ethnicity or who preferred not to answer (of these, 821 [89.9%] self-identified as Latinx). The SARS-Cov-2 positivity rate was 14.5% for Latinx individuals and 13.8% for non-Latinx individuals. We next tested the effectiveness hypothesis for total Latinx sample tests collected and proportion of the Latinx populace tested. Results of the multilevel negative binomial and linear regressions are presented in Table 2. Incident rate ratios (IRR) greater than 1 indicate a greater likelihood of a sample test being for a Latinx individual, and IRRs of 1 or lower indicate a lower likelihood. The effectiveness hypothesis was supported. The IRR coefficient for the intervention contrast showed that intervention sites tested 3.84 (95% CI, 2.47-5.97) Latinx participants for every 1 tested at a control site, controlling for site characteristics and time-varying COVID-19 variables. The intervention effect represents a medium to large effect size (Cohen *d* = 0.74).^28^ Tests of time-varying random effects vs fixed effects supported specification of random effects for time-varying covariates (change χ^2^_5_ = 88.49, *P* < .001). Estimate evaluation also indicated that the model potentially underfitted zeros because of zero inflation. A post hoc sensitivity analysis for a zero-inflated negative binomial^29^ adjusting for both overdispersion and zero inflation obtained identical substantive findings. Among site characteristics, higher community nativity was associated with a 38% lower likelihood of Latinx persons tested. As indicators of the pandemic climate, not surprisingly, as 1 week lagged newly confirmed cases increased over time, there were 1.75 more individuals tested per all testing events (95% CI, 1.35-2.27; *P* < .001). Conversely, there was a 1% decrease in the likelihood of a Latinx test for every 1% increase in county vaccine coverage, which ranged from 3% to 60%.

**Table 1.** Repeated Testing Event Outcomes and Site Demographic Characteristics by Group Condition and Total Sample

Variable	Total sample (394 events, 33 sites)		*Promotores* (212 events, 18 sites), mean (SD)	Outreach as usual (182 events, 15 sites), mean (SD)
	No. (%)	Mean (SD)		
**Testing event level**				
Sample tests	1851 (100)	4.70 (8.02)	5.72 (9.77)	3.51 (5.07)
Sex				
Male	832 (44.9)	2.11 (3.83)	2.49 (4.69)	1.67 (2.42)
Female	995 (53.8)	2.53 (4.34)	3.15 (5.18)	1.80 (2.93)
Nonbinary or other	24 (1.3)	0.06 (0.36)	0.08 (0.45)	0.04 (0.19)
Race and ethnicity				
Latinx	919 (49.6)	2.33 (3.13)	3.03 (3.44)	1.52 (2.48)
Proportion of Latinx populace ×100	NA	0.30 (0.53)	0.40 (0.66)	0.19 (0.30)
American Indian or Alaska Native	85 (4.6)	0.22 (0.64)	0.28 (0.75)	0.14 (0.47)
Asian	26 (1.4)	0.07 (0.35)	0.05 (0.29)	0.09 (0.41)
Black or African American	16 (0.9)	0.04 (0.24)	0.04 (0.25)	0.04 (0.23)
Native Hawaiian or Pacific Islander	8 (0.4)	0.02 (0.16)	0.02 (0.15)	0.02 (0.17)
White	746 (40.3)	1.89 (5.27)	2.23 (6.74)	1.51 (2.67)
>1 Race or ethnicity	57 (3.1)	0.14 (0.55)	0.19 (0.69)	0.09 (0.33)
Unknown or not reported	913 (49.3)	2.29 (3.34)	2.87 (3.59)	1.62 (2.90)
Vaccine event	16 (4)	0.04 (0.20)	0.04 (0.20)	0.04 (0.19)
Weekly county-level data				
Log lag COVID-19 cases	NA	162.62 (168.19)	161.97 (181.85)	163.38 (151.22)
Lag percentage of vaccination	NA	27.57 (15.87)	27.74 (16.01)	27.39 (15.75)
**Total site level**				
Census tract				
Total populace	NA	5657.00 (1708.87)	6002.50 (1767.94)	5242.40 (1594.31)
Latinx populace	NA	1296.58 (1135.37)	1323.00 (1070.34)	1264.87 (1246.33)
Native-born populace	NA	5045.88 (1518.29)	5358.72 (1607.10)	4679.47 (1362.34)
Income inequality	NA	0.42 (0.05)	0.42 (0.06)	0.42 (0.04)
Median age	NA	39.52 (7.21)	38.80 (5.05)	40.38 (9.29)
Repeated events held	NA	11.94 (2.72)	11.78 (3.17)	12.13 (2.13)
Site type^a^				
College	1 (3.0)	NA	1 (5.6)	0
Commercial	4 (12.1)	NA	2 (11.1)	2 (13.3)
Community center	1 (3.0)	NA	0	1 (6.7)
Place of worship	7 (21.2)	NA	5 (27.8)	2 (13.3)
Public park	1 (3.0)	NA	1 (5.6)	0
School	15 (45.5)	NA	6 (33.3)	9 (60.0)
School district office	4 (12.1)	NA	3 (16.7)	1 (6.7)

Abbreviation: NA, not applicable.

^a^ Values for intervention and control site types are presented as number (percentage). Positivity rates were not available by group condition or at the individual level because of Clinical Laboratory Improvement Amendments compliance and consenting requirements. Rates are estimated here in the aggregate.

**Table 2.** Estimates and Variance for Number of Latinx Individuals Tested and Proportion of Latinx Populace Tested Regressed on Multilevel Predictors

Variable	No. of Latinx individuals tested multilevel (negative binomial)			Proportion of Latinx populace tested ×100 (multilevel regression)		
	IRR (95% CI)	*P* value	Estimate (95% CI)		*P* value
Fixed effect					
Intercept	0.79 (0.46 to 1.34)	.38	0.27 (0.07 to 0.48)		.009
*Promotores*	3.84 (2.47 to 5.97)	<.001	0.28 (0.11 to 0.45)		.001
Vaccine offered	1.94 (1.08 to 3.48)	.03	0.25 (0.06 to 0.44)		.01
Census tract					
Latinx populace	1.03 (0.99 to 1.05)	.06	–0.00 (–0.01 to 0.01)		.37
Native-born populace	0.62 (0.48 to 0.80)	<.001	–0.10 (–0.20 to –0.01)		.04
Income inequality	0.95 (0.79 to 1.14)	.59	0.01 (–0.07 to 0.08)		.84
Median age	1.29 (0.99 to 1.67)	.06	0.09 (–0.01 to 0.20)		.07
Time-varying random effect					
Log lag COVID-19 cases	1.75 (1.35 to 2.27)	<.001	0.15 (1.39 to 2.30)		.01
Lag vaccination coverage	0.99 (0.98 to 0.99)	.004	–0.004 (–0.007 to –0.001)		.02
Explained variance					
Fixed-effects *R*^2^	.29	NA	.18		NA
Fixed- and random-effects *R*^2^	.49	NA	.57		NA

Abbreviations: IRR, incident rate ratio; NA, not applicable.

**Figure 2.**
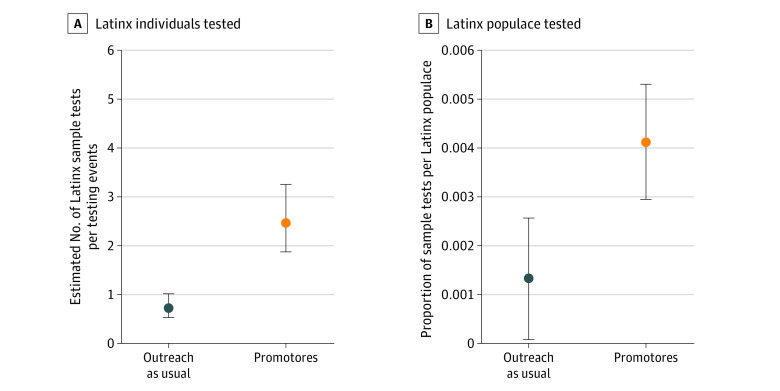
Intervention Effect Sizes for the Primary Outcomes A, Model-based estimates of *Promotores de Salud* intervention effect on predicted numbers of Latinx individuals tested per testing event (incident rate ratio = 3.84; 95% CI, 2.47-5.97; Cohen *d* = 0.74). B, Community health promoters intervention effect on proportion of Latinx populace (33 sites, 394 testing events, and 1851 individuals; effect size = 0.53). Error bars indicate 95% CIs.

**Figure 3.**
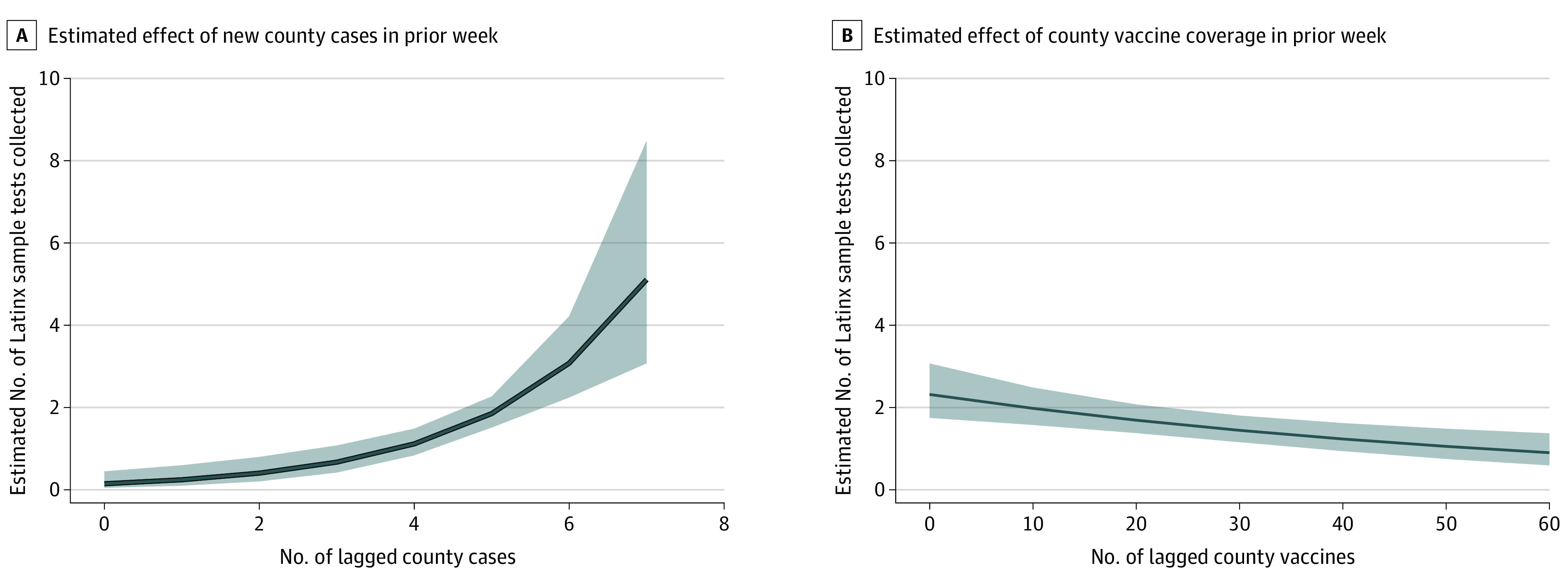
Time-Varying Effect of COVID-19 Transmission and Vaccination Coverage A. Model-based independent time-varying effects of new COVID-19 county cases in the prior week. B. Effect of percentage of county vaccine coverage in the prior week on the estimated numbers of Latinx individuals tested per testing event (33 sites, 394 testing events, and 1851 individuals). Shaded areas indicate 95% CIs.

The intervention was associated with a 0.28 increase in the proportion of the Latinx populace being tested relative to control sites for the dependent variable scaled as the proportion of the Latinx populace ×100, or a 0.003 proportion of the raw populace count. The use of a standardized scaling of the proportion of Latinx individuals showed that the relative percentage increase was 0.53 (95% CI, 0.21-0.86) in the intervention sites relative to controls, for a medium effect size. Data also demonstrated a greater proportion of Latinx persons among all those tested (0.18; 95% CI, 0.06-0.31; *P* = .004). The model-based intervention effect size for primary outcomes is displayed in Figure 2, and the time-varying effect of COVID-19 transmission and vaccination coverage is shown in Figure 3. Results provide causal evidence of program effectiveness in reaching and testing a disproportionately affected underrepresented population.

## Discussion

This study presents causal evidence that culturally tailored community outreach can increase SARS-CoV-2 testing rates among Latinx community members. The OSJP evaluation obtained medium to large effect sizes for increasing testing rates among the Latinx population in the state of Oregon relative to wait-listed county outreach practices as usual.

Explanatory factors accounting for health disparities and drivers of health inequity must be understood within the context of systematic historical and ongoing discrimination, chronic stress, and compromised immunologic functioning.^4^ As Webb Hooper and colleagues^4^ argued, given documented disparities, including differential access to health insurance, health care, testing, and quality hospital care, there is an obligation to address these predictable consequences with evidence-based and culturally responsive interventions. To directly address health disparities, health scientists need to think outside the comfort zones of our clinics, laboratories, hospitals, and universities to effectively engage and meet the collective needs of our communities, just as we would prescribe correct medication to treat an individual.^30,31^

The OSJP project applied such a model to provide experimental evidence of these principles. Effective participatory community engagement required building and maintaining key partner relationships across the duration of research and testing activities. Such approaches work with the Latinx community instead of developing interventions for the Latinx community and are shown to be effective for mitigating other health disparities.^32,33^ Engaging community partners was not without challenges. Before vaccine availability, some community partners understandably objected to randomization and wait-listed sites. Responsivity to concerns with county officials and CBOs helped establish trust to complete the primary aim of experimentally evaluating the outreach program before releasing all sites to *Promotores de Salud*. Through relationship maintenance with stakeholders, we were also able to establish sustainability through state-requested contracted services for ongoing testing sites, independent of the experimental trial. All efforts were guided by and supported by a collaboration with a community and scientific advisory board that included Latinx physicians, researchers, and community leaders.

### Strengths and Limitations

Strengths of the study included an experimental design with site randomization within counties to minimize threats to internal validity. Data showed no comparable differences by group condition site characteristics. Time-varying COVID-19 transmission and vaccine data were lagged to meet temporal assumptions of causal inferencing. Limitations included an overall range of significant residual variation, suggesting that unmeasured factors and factors such as implementation quality may be associated with this variation (eg, fidelity of implementation and adherence to outreach strategies). The study covered a large portion of counties in Oregon and was generally representative of state demographic characteristics; generalizability for other underrepresented groups, however, was limited. The study was also geographically dispersed in attempts to cover rural and urban areas in a largely rural state. Beyond the scope of this study, a cost-benefit analysis would benefit the planning and resourcing of similar implementations of the *Promotores* model. We have established sustainability and cost sharing for ongoing testing outside the randomized study scope.

## Conclusions

In this cluster randomized trial, the *Promotores de Salud* outreach intervention had a larger number of Latinx individuals tested per repeated testing events. Moreover, the intervention group saw an increase in the proportion of the Latinx populace tested for each testing site’s census tract area. Controlling for time-varying COVID-19 factors, the intervention’s outcomes represented medium effect sizes. Findings suggest that culturally tailored outreach can be implemented or adapted to serve future needs for community engagement during a health crisis or to address ongoing health disparities. Outreach delivered by bilingual and bicultural community health promoters included (1) use of a facilities-location procedure to reduce drive time, (2) Spanish-language materials, (3) not requiring health insurance or state-issued identification, (4) not requiring physician orders, (5) not charging fees, and (6) allowing onsite walk-up registration. Other factors necessary for effective sustained community engagement included formation and maintenance of a community scientific and advisory board as partners in the implementation work. Developmental work must be iterative in partnership with community leaders, including pilot testing of all materials and protocols. Community trust should not be expected but earned.

## Notes

Supplement 1.
Trial Protocol

Supplement 2.
Data Sharing Statement
