# Supplementary material for: Effectiveness of a COVID-19 Testing Outreach Intervention for Latinx Communities: A Cluster Randomized Trial
Source: JAMA Netw Open. 2022 Jun 16;5(6):e2216796. doi: 10.1001/jamanetworkopen.2022.16796 (PMC9204550; doi:10.1001/jamanetworkopen.2022.16796)
Supplement: Supplement 1. — Trial Protocol [file jamanetwopen-e2216796-s001.pdf]

Research Plan

IMPORTANT: When completing this outline, please use the **Research Plan Guidance** for the content necessary to develop a comprehensive yet succinct Research Plan. Using the guidance to complete this outline will help facilitate timely IRB review.

Study Title: Scaling Up SARS-COV-2 Testing to Serve Latinx Communities

Protocol Number: 10032020.002

Principal Investigator: Leslie D. Leve

A. Introduction and Background

SARS-CoV-2 is a high contagious virus that has infected millions of Americans and is disproportionately impacting the Latinx population. Nationally, Latinx people are hospitalized with COVID-19 infections at four times the rate of Whites. This disparity is notable in Oregon, where Latinxs represent approximately 44% of COVID-19 cases, while representing only about 13% of the state's population. Therefore, there is an urgent need to reach Oregon's Latinx community with public health and prevention messages that are culturally-relevant, address community concerns, and are delivered through appropriate information channels in order to increase testing rates and ultimately, reduce the spread of infection and mortality rates.

The overall goal of the proposed research is to implement a culturally-tailored community outreach and testing program to increase the reach, access, uptake, and impact of testing in Latinx communities in Oregon. Our project has received funding to provide SARS-CoV-2 testing across the state of Oregon from two sponsors: National Institutes of Health (NIH) (EPCS #30680 and EPCS #29424) and Oregon Health Authority (OHA) (EPCS #31138). NIH funded events include a individual-level data collection component (participant survey) to address study aims. OHA funded events will not include any individual-level data collection, and will only collect de-identified protected health information.

B. Specific Aims/Study Objectives

The overall goal of our proposed research is to implement a culturally-tailored community outreach and testing program to increase the reach, access, uptake, and impact of testing in Latinx communities in Oregon. This project will fully integrate with the NIHRapid Acceleration of Diagnostics (RADx) consortium and its Coordination and Data Collection Center (CDCC). With guidance and leadership from our Latinx Community and Scientific Advisory Board, we will launch the proposed testing program which will result in 36 community testing sites to serve Latinx communities. For the proposed research activities, we will test the following questions:

1) Do culturally-informed interventions ('Promotores') increase test rates and health behaviors?
   a. Community-level intervention to increase participation in testing: Evaluate whether 'Promotores de Salud,' a culturally-tailored outreach intervention that includes psychoeducation, information support, and motivational strategies to address barriers, results in more tests completed and more sites testing at full capacity, as compared to basic community-level outreach services.
   b. Individual-level booster intervention to change future health behavior: Examine whether a brief, individual-level 'Promotores de Salud' intervention held during a testing event (compared to distribution of a pamphlet only) results in greater use of strategies that reduce transmission at the community and individual level. Individual moderators (e.g., substance use, perceived threat, health literacy, social determinants) will also be examined. We further hypothesize that this intervention will increase future test rates through repeat testing visits and

Research Compliance Services
Approved 11/19/2021 - 9/30/2022

through 'snowball' recruitment of friends and family to attend future testing events.

C. Methods, Materials and Analysis

Overall Study Design: We will partner with community organizations to administer SARS-CoV-2 tests at organized, planned testing events located in communities across Oregon. Sample collection will occur in counties across Oregon and we will collaborate with county public health agencies to determine sites. Samples will be collected using services as usual using a standardized intake form and standardized, EUA authorized sample collection approaches. Data used to evaluate the efficacy of interventions will involve de-identified participant data at the level of the testing site. This study is a randomized clinical trial to test whether 'Promotores,' community health workers who deliver culturally-informed set of outreach, education, and engagement interventions, will result in an increase in testing utilization and health behaviors in Latinx communities.

Randomization will occur at the site level such that 18 sites will be randomized to receive the Promotores intervention and 18 will be randomized to receive services as usual. Services as usual include outreach by county public health and community-based organizations (fliers, radio ads, social media, etc.). The promotores intervention includes targeted outreach to Latinx community members and educational direct interactions with participants and distribution of a pamphlet, with the goal of increasing health behaviors and encouraging additional/future testing. The UO will provide support to community-based organizations to employ and train these promotores to assist with recruitment. The promotores will include Spanish speakers, across cultural contexts, living and working in, and intricately connected to, the target community.

- **Outreach as usual description**
  - Basic outreach materials document (flyers, social media posts, radio announcements, etc.)
  - Flyer to promote health behaviors
  - These documents may be modified or not used at all by community stakeholders as they may have other documents they are already using for outreach and health education—these materials are intended to be used for counties who do not have existing or preferred materials
  - See 04c.Services as Usual Materials
- **Promotores intervention description**
  - Basic outreach and flyer to promote health behaviors plus the outlined outreach and health behaviors promotores approaches
  - Promotores training will be provided by the study team
  - See 4d. Promotores Training Outline

Data points to assess testing site utilization and the health behaviors will include the following:

1. **De-identified protected health information** (from intake forms) from those who engaged in testing (age 3 and older)
   a. After testing occurs, researchers will have access to de-identified data prepared by the UO's CLIA laboratory. The data will include four protected health identifiers, site zip code, participant zip code, testing date, and SARS-CoV-2 test result. It will also include demographic data this is not protected health information such as CDC age range, race, and ethnicity. These data will be used to assess outreach activities aimed at increasing participation of Latinx community members in SARS-COV-2 testing. Primary outcomes will be site-level testing rates of Latinx individuals.
   b. In addition, the study team will record and have access to a list of testing site addresses and testing date, that will be merged with the aggregate file provided by the CLIA laboratory.

Research Compliance Services
Approved 11/19/2021 - 9/30/2022

2. **Individual level survey data**, collected at NIH-funded testing events from a subset of 3,600 adult participants (age 15 and older).
    a. Surveys will contain information including demographic information, including address, phone number, and email. This is being collected to link the HIPAA data that we are collecting from the UO CLIA laboratory, in addition to allowing researchers to see if individuals are getting tested in close proximity to their residence. Researchers are looking at population density of Latinx individuals in an area compared to the proportion of Latinx people who attend the testing events. Additionally, the survey will include information about health behaviors to prevent the spread of COVID-19. These data will be collected using an electronic survey that participants can complete on their smartphone, or using an iPad provided on site. If preferred by the participant, the survey may be completed on paper. If the participant prefers or as is needed, the survey items will be read by a field team member, who is bicultural and bilingual. Surveys will be available in Spanish and in English.
    b. Individuals who are willing to participate in the survey will be asked in the first survey how they prefer to complete the follow-up survey. They will be able to select to receive a text message with a follow-up survey link, email with a follow-up survey link or a paper survey by postal mail with a pre-paid envelope to return the survey, one month later. For participants who prefer, the one-month survey will be conducted by telephone. Each survey will take approximately 30 minutes to complete.
    c. As part of standardized testing protocols, participants will complete an intake form. As part of the consent and assent process, they will provide consent or assent to share their identifiable protected health information with the research team. Their intake data will be linked to the survey data.
    d. Survey data will be collected in-person at testing sites, requiring direct interaction with participants.
3. **Anonymous participant interview.** A random set of individuals will be asked to participate in the anonymous interview following testing. Participants who verbally agree to this component will be directed to meet with a study staff member at a testing event after they complete the testing event activities. A study staff member will verbally consent the participant using the consent script. This aspect of the study is now complete.
4. **Promotores activities.** In addition to non-human subjects activities (e.g., reporting on training, outreach activities and interactions with participants), promotores will be asked to complete two questionnaires to provide some demographic information, information about their personality traits (e.g., conscientiousness), and feelings about participating as a Promotor/a on this project. They will be asked to complete an electronic informed consent before participating in the interviews.
    a. The first survey will directly follow the promotores training and will ask basic demographic information and information on their personality traits.
    b. The second interview will be in the format of a focus group and will ask questions about their experience as a promotor/a and their perspectives on the intervention.
5. **Community Based Organization general outreach activities**. The executive director of each CBO that receives promotores funding will provide a monthly report on the general outreach strategies conducted by the organization. This is a condition of receiving funding, but reports will be included in research reports such as peer reviewed journal articles.

D. Research Population & Recruitment Methods

We anticipate the sample to be comparable with community demographics in each of the testing regions within the counties participating in this study. As we are specifically conducting outreach to encourage Latinx individuals to attend testing events, we expect the overall study population to be 85% Hispanic and 15% non-Hispanic, with 84% of individuals identifying as Caucasian in both ethnic groups and the remaining 16% identifying as African American, Asian, Pacific Islander,

Research Compliance Services
Approved 11/19/2021 - 9/30/2022

American Indian/Alaska Native, or more than one race. We expect a slight excess of female participants, based on the demographics in the standing population.

1. De-identified protected health information participants will include data from all testing sites participants which will include individuals age 3 and older. We expect approximately 900 people per week will attend a testing event and we will assess outcomes over 40 weeks, thus we expect between 32,000- 36,000 individuals will be included in the testing. Individuals will not be directly recruited to provide these data.

2. Individual level survey data will include participants who are (a) age 15 years and older; (b) attending an NIH-funded testing event. Exclusion criteria include: (a) individual is unable to understand Spanish or English or another language translated by a qualified translator at a 5th grade level (participants who cannot read will have the consent or assent form read to them); (b) individual discloses that they previously enrolled in the research project. We anticipate up to 3,600 adult individuals will provide survey data. Up to 1,800 individuals will be individuals who had received the promotores intervention and up to 1,800 will have received services as usual. Recruitment will occur at the testing site, by a study research assistant. A standardized recruitment script (see recruitment information document) will be used and will include eligibility questions. Participants will be compensated with a $30 gift card for each survey they complete (one at baseline, one at one month follow-up for a total of $60 in gift card renumeration available). To recruit participants, we will also add the following text to testing event flyers circulated by our community partners '$30 gift cards available for those eligible to participate in a research survey.'

3. Anonymous participant interview will be collected from a sub-set of randomly selected individuals at NIH-funded testing events who are age 18 years or older. We will invite approximately 15 individuals to participate in a brief anonymous interview (5-8 open-ended questions) with a study staff member at our testing events. Anonymous interview data will assist with informing the study about individual behaviors related to attending testing events. We will audio record these interviews for transcription. All audio recordings will be destroyed after transcription activities are complete. A standardized recruitment script will be used, which will provide information about the purpose of the interview and permission for audio recording. Individuals will be compensated with a $15 gift card for completion of this interview. These anonymous interviews will not be used as research data, and are only to provide feedback on the testing events. This aspect of the study has now been completed.

4. Promotores activities. Promotores will be recruited after receiving contact information from the community-based organization employing each promotor. They will be invited to participate in the promotores training and, for NIH-funded testing events, they will be asked to complete the informed consent and survey asking about their demographics and personality during the course of the promotores training. Promotores will also be invited to participate in a focus group interview about their experiences delivering the intervention and opinions on the project and content delivery. There is no monetary incentive for participation. The only inclusion criteria will be that the individual must be a promotor funded by the present study.

5. Community Based Organization general outreach activities. A monthly email will be sent to the executive director of each CBO participating in the NIH-funded testing events to complete a survey on general outreach activities. Completion of the survey is part of the requirements for receiving CBO funding.

For data analysis, we will employ standard normal theory generalized linear modeling analysis of covariance fractional factorial analyses using pre-post assessment scores for site and individual level outcomes.

Research Compliance Services
Approved 11/19/2021 - 9/30/2022

E. Informed Consent Process

1. De-identified protected health information participants. We will not obtain informed consent for using de-identified testing data. Informed consent will not be obtained as processes and procedures will occur as usual for sample collection and we will only receive limited protected health information from the CLIA laboratory. We are requesting a complete waiver of informed consent for this aspect of the research.

- The research involves no more than minimal risk to the subjects because the data collected will not be identifiable
- The research could not be practicably carried out without the waiver, because asking participants to complete the informed consent process would substantially increase the length of time required for testing and would result in fewer resources available for testing
- While the research uses HIPAA identifiers, it does not include identifiable patient information (see HIPAA 4a)
- The waiver will not adversely affect the rights and welfare of the subjects as data provided to the study team will not be identifiable
- There is not a need to provide additional pertinent information after participation

2. Individual level survey data We will use a standardized consent form to consent participants 18 and older at NIH-funded testing events. Individuals between the ages of 15-17 at NIH-funded testing events will use a standardized assent form to agree to participate. All individuals will read the consent or assent form and will be asked if they have any questions. For individuals who do not speak English or Spanish, a translator may be available in some instances. Individuals over 18, who cannot read at a 5th grade level will have the consent form read to them. When appropriate, translators will be available for individuals in the Latinx community who speak languages other than English or Spanish, such as Mam. Children will never be used as translators.

The assent form is at a 6th grade reading level and is in plain language. Any questions will be answered by the research staff.

We are requesting a waiver of parental consent to allow individuals between the ages of 15-17.99 to participate in this research study. The age of medical consent in Oregon is 15, so COVID-19 testing is available at our events to individuals 15 years of age and older without parental consent. Because of this, many individuals between the ages of 15-17.99 come to COVID-19 testing events without a parent present. We are requesting to seek their assent, and not consent of their guardian.

- The research involves no more than minimal risk to the subjects because the data collected will not be identifiable
- The research could not be practicably carried out without the waiver, because asking the guardian of participants age 15 – 17.99 to complete the informed consent process would substantially increase the length of time required for testing and would result in fewer resources available for testing
- The waiver will not adversely affect the rights and welfare of the subjects as data provided to the study team will not be identifiable
- There is not a need to provide additional pertinent information after participation

Participant consent or assent will include consent or assent to share protected health information from the University of Oregon's CLIA certified laboratory, with the study team, so we can link testing intake data with participant survey data. During the informed consent and assent process, participants will be informed that their personally identifiable information is being collected to link testing intake data with their survey data and to be able to understand how well we are reaching people who identify as Latino/a/x in a community.

Participants will be assured that their participation in the study is voluntary and that if they choose to participate, they can change their minds at any time. They will be informed about potential benefits

Research Compliance Services
Approved 11/19/2021 - 9/30/2022

and harm. They will be informed that their decision about whether to participate will not alter the medical care, or social services they will receive at any time from the county partners and that they will not be denied services of any kind. Participants over 18 years old will be asked to sign an informed consent form that explains the project, their rights, and how the data will be used in the future. Participants between the ages of 15-17 will sign an assent form that explains the project, their rights, and how the data will be used in the future. A phone number and email address linking participants to Spanish-speaking and English-speaking members of the research team will be provided so participants can contact project staff if they have any questions about the consent or assent form. Translation services may be leveraged if a participant does not speak either language. Research assistants will be trained in recruitment procedures and have specialized training in how to handle any exceptions. The random assignment component of the study will be carefully explained and it will be made clear that the randomization will not affect the SARS-CoV-2 testing procedure or the reporting of results. We have used similar consent and assent procedures in many prior intervention research studies and have successfully recruited large numbers of participants in community agencies.

At the time of consent or assent, the research staff will review the consent or assent form with the participant before they sign it to ensure that the participant is clear about data will be obtained by National Institutes of Health and Duke Clinical Research Institute versus the UO. The research staff will also clarify that the identifying information that the participant includes in the CDE survey will be used by National Institutes of Health (NIH) and Duke Clinical Research Institute (DCRI) to link to other data such as Medicaid data, though once linked the data will be de-identified. The research assistant will also clarify that Medicaid and other data to which NIH/DCRI links will not be used by UO. Participants will be presented with the consent or assent to participate in the survey. Following that agreement, by obtaining a typed name, participants will be invited to link intake data and test result to the survey and core elements of a HIPAA authorization will be presented. A signature will be collected for the HIPAA Authorization (typed and finger drawn). Refusal to allow linking of intake and test result is not an exclusion criterion.

An informed consent form and assent form will be posted on a Federal Web site (e.g., clinicaltrials.gov) after the clinical trial is closed to recruitment, and no later than 60 days after the last study visit by any subject.

3. Anonymous participant interview. We will not obtain documentation of informed consent from participants to complete this interview. Instead participants in NIH-funded testing events will be asked to verbally agree to participate in the anonymous interview. Therefore, consent will be solicited from participants verbally, and there will not be documentation of their consent. Thus, we are requesting a waiver of documentation of consent for this component of the research, to allow for a verbal consent process. Verbal consent to participate will be received prior to starting the interview. This aspect of the study is now complete.

- The research involves no more than minimal risk to the participants because the data collected will not be identifiable
- The waiver will not adversely affect the rights and welfare of the participants as data provided to the study team will not be identifiable
- There is not a need to provide additional pertinent information after participation
- Participants will be asked to verbally consent to completing the interview, after 1) being informed that this is a research study; 2) given a description of foreseeable risks and discomforts of participating; 3) provided contact information for the Project Coordinator, should they have questions.

4. Promotores activities. We will use standardized consent forms to consent participants in NIH-funded testing events. All individuals will read the consent form and will be asked to reach out to the

Research Compliance Services
Approved 11/19/2021 - 9/30/2022

study team if they have any questions. The consent forms will be programmed in Qualtrics. The Qualtrics links will be provided to Promotores by email. Promotores will sign their name, type their name, and provide the date to indicate informed consent. Promotores will be invited to share information about their demographics and personality, as well as participate in a focus group to share their experiences and opinions on the intervention. Sharing this information is optional.

5. Community Based Organization general outreach activities. Consent is not required for this activity because we are collecting data on the organization activities, rather than individual behaviors or impressions.

F. Provisions for Participant Privacy and Data Confidentiality

As a requirement from our funder, de-identified data will be provided to the Coordination and Data Collection Center (CDCC). This is referred to as a data library in the consent and assent. Data will not be identifiable and will be shared using secure processes and procedures as designated by the CDCC.

1. Privacy

De-identified protected health information participants. Potential participants will be identified by community partners (e.g., county public health agencies or a local health care provider) and the research team will not have access to their information, other than the data described in the methods, materials and assessment section. Participants will be tested in an environment that meets the patient privacy standards of the care provider (e.g., behind a partition, or in their vehicle at a drive-up event).

Individual level survey data. During the intervention, it will be up to participants to disclose vulnerable information about their previous experiences. Though testing will occur in public spaces, as is common in current pop-up testing across Oregon, efforts will be made to ensure that participants will be recruited, consented or assented, and tested in a private environment (e.g., behind a partition, facing away from others, in their vehicle, etc). Participants will only disclose contact information they feel comfortable disclosing, which may include email, phone number, or physical address.

Anonymous participant interview. Participants will not be asked to provide any identifiable information during the anonymous participant interview. In addition, efforts will be made to ensure participant confidentiality. For example, the interview will be conducted out of earshot from others. This aspect of the study is now complete.

Promotores activities. While promotores must participate in the knowledge assessment post training to determine adequate skill and knowledge to perform their role and must complete fidelity checklists to ensure program integrity, they will be able to elect to respond to questions asking for information about their personal characteristics or experiences related to the training.

Community Based Organization general outreach activities. CBOs will never be identified by name in any external scientific reports, however CBO contracts are federally funded and thus publicly available so there is potential that CBOs could be identified.

2. Data Disposition

De-identified protected health information participants. Participant research data and samples will be collected without individual identifiers. Data will be maintained by the research study team. Barcode identifiers will be used to link survey data to biospecimens. The research team may be briefly in possession of identifiable information while transporting diagnostic samples and only team members listed on the protocol will be involved in the transportation of diagnostic samples.

Research Compliance Services
Approved 11/19/2021 - 9/30/2022

Individual level survey data Identifiable participant information will be collected in Qualtrics or RedCap during each survey. Following data collection, identifiable data will be removed, and a unique identifier assigned for all analyses. Protected health information from the sample collection intake form will be collected and stored by the University's CLIA laboratory. Only participants who provide consent or assent to share identifiable data with the study team will have their identifiable data shared with the study team. Disposition of email, phone number, or physical address will include keeping these data separate from the client survey data for analysis.

Anonymous participant interview. This component does not include the collection of individual identifiers. Audio recordings of participant interviews will be maintained on the secure server at the Prevention Science Institute (see confidentiality). Immediately following transcription, audio files will be destroyed.

Promotores activities. Identifiable participant information will be collected in Qualtrics or RedCap during each survey. Following data collection, identifiable data will be removed, and a unique identifier assigned for all analyses.

Community Based Organization general outreach activities. CBOs will never be identified by name in any external scientific reports, however CBO contracts are federally funded and thus publicly available so there is potential that CBOs could be identified.

3. Confidentiality

All data points. All de-identified research survey data will be stored at the Prevention Science Institute using standard security techniques (password protected file folders on the University's Prevention Science Institute secure server). Data from Qualtrics or RedCap will only be accessed using secure wifi. GC3F will store the viral RNA data on Talapas. Any sharing of de-identified viral RNA or survey data will occur on shared drive (such as One Drive) that is password protected.

Storage of data will be stored electronically on a private server and will be directly uploaded only while using secure wifi. If the data must be transferred, they will be transferred using a secure client server. Study researchers and the students they supervise may be granted access to the de-identified data after signing a data use agreement, in order to complete analyses. Records will be kept for up to 3 years after the study has been completed.

In addition to our study's database and data storing procedures within the UO, data collected for the RADx-UP project that will be shared with the RADx consortium will be uploaded through a secure client server and maintained by staff at the Duke Clinical Research Institute (DCRI). Identifiable data will be shared with DCRI when permission is given in the informed consent form or assent form. Participants are given the option to share identifiable information with the DCRI on the informed consent form or assent form. Agreeing to share identifiable data with the DCRI is not a requirement for participants to be enrolled in the study. The DCRI manages data from all studies participating in the RADx Program. The DCRI maintains data in two separate databases, one that contains identifiable information and one contains de-identified information.

Individual level survey data and promotores data. The only individuals with access to identifiable participant information on Teleform, Qualtrics or RedCap will be those who have the appropriate human subjects training who need access to the data to complete their assigned role on the research team. The code and key with participant contact information will be retained in a locked file on the Prevention Science Institute (at University of Oregon) server with permissions granted to only to the study project coordinator and data manager.

Research funded by NIH automatically has a Certificate of Confidentiality associated with it. This is added protection against forced disclosure of research information in circumstances of subpoena.

Research Compliance Services
Approved 11/19/2021 - 9/30/2022

G. Potential Research Risks or Discomforts to Participants

De-identified protected health information participants. Potential risks and discomforts involved in participation include (1) possible violation of confidentiality. These risks are unlikely. All records obtained from the CLIA laboratory will be kept strictly confidential. Identification numbers will identify participants' data, not their names. Project staff will receive training on confidentiality, including data collection, data management, and reporting procedures.

Individual level survey data. Information risks, psychological risks, and social risks may occur as the result of participating in this study. Information risks include loss of privacy and/or breach of confidentiality. Since data will be collected using only private wifi (except when participants are not connected to secure wifi if they use their own device) and will be stored on a secure server, it is unlikely that loss of privacy will occur.

Psychological risks may occur such as guilt or loss of self-esteem while receiving the health intervention. It is likely that both the treatment as usual and the treatment conditions will improve sense of responsibility to engage in health behaviors without inducing guilt and improve self-esteem but not everyone receives treatment uniformly. Participants will be encouraged to seek services if they are in need of additional support.

Social risks may occur as a result of receiving services in public. Specifically, participants might feel uncomfortable engaging in the intervention if they do not have a private space to participate. This risk may be diminished by the fact that half of the participants at the intervention site will be offered the intervention, regardless of their participation in the research survey.

All risks will be communicated in the informed consent or assent process. Our consenting and assenting process informs participants that the information that they provide will be shared with NIH and the RADx program who will collaborate to combine data that are collected in a standardized way across the group of studies. In addition, participants are reminded that all data will be de-identified prior to sharing with anyone outside of our study or outside of the RADx-UP program. Participants have the option to agree to let the DCRI have access to identifiable information (i.e. name, address, contact information, and date of birth) by providing their initials on the informed consent or assent form.

The protocol involves intervention. The intervention includes a promotores or community navigator intervention that aims to increase testing utilization and health behaviors. This will involve outreach by promotores present at testing sites. Promotores will provide health information which includes information from the Centers for Disease Control that has been vetted by county public health professional. This research does not pose any additional risk over the services provided as usual since some communities provide similar interventions.

The funding agency required a Data Safety Monitoring Plan for this research at the time of funding proposal. A copy of the DSMP submitted to the funder is attached.

There is no established Data and Safety Monitoring Board/Committee (DSMB/C) as noted in the DSMP.

Anonymous participant interview. There are no physical risks for the individuals who choose to complete this interview, however, some participants may experience discomfort when asked behavior related questions. Participants who feel uncomfortable during any part of the interview have the option to skip any question. This aspect of the study has been completed.

Promotores data. Information risks and psychological risks may occur as the result of participating in this study. Information risks include loss of privacy and/or breach of confidentiality. Since data will be collected using only private wifi (except when participants are not connected to secure wifi if they use their own device) and will be stored on a secure server, it is unlikely that loss of privacy will occur. All risks will be communicated in the informed consent and assent process.

Research Compliance Services
Approved 11/19/2021 - 9/30/2022

H. Potential Benefits of the Research

The overall risk involved in the project is relatively minor, given the goals of the research. Participants would face similar levels of risk sharing their information with a clinic or other outdoor testing event in order to be tested for SARS-CoV-2 infection. Participants will benefit by receiving a free SARS-CoV-2 diagnostic test and educational information that could improve their health behaviors and reduce transmission of SARS-CoV-2 in their communities and around Oregon. Thus, others in the community would benefit from general participation if participants are identified as positive who previously did not know they were infectious, and if participants actively alter their health behaviors, whether infectious or not.

Individuals may receive benefits from participating in this research such as improvements in health behaviors and reduced likelihood of contracting and spreading COVID-19.

By participating in this research, the research team will be able to learn whether the promotores intervention would be generally useful over services as usual (a flyer about health behaviors).

I. Investigator Experience

1. Investigator Qualification

MPI: Leslie Leve, Ph.D., Leve is the Alumni Faculty Professor in the College of Education at the University of Oregon. She has extensive expertise in directing and managing multicomponent and multi-site projects, including her role as Principal Investigator of an ECHO cohort award from NIH which contains three cohorts of families, coordination with her cohort co-investigators across the U.S., and coordination with the larger ECHO consortium of more than 30 awardees. She co-chaired the Data Sharing working group for ECHO and serves on its Return of Results workgroup. On her P50 Center of Excellence award, she directs the Administrative Core, and received a HEAL supplement to focus on web-based platforms for the prevention of substance use in adolescent and young adult populations. Both ECHO and HEAL have extensive data harmonization and data sharing requirements, which Leve's projects have fully met. She holds national and local leadership roles that require outstanding research and leadership skills, including her past role as President of the Society for Prevention Research, and her current roles as Associate Vice President for Research at the University of Oregon and Associate Director of the Prevention Science Institute. She is an accomplished scholar, with over 170 peer reviewed publications focused on research in partnership with community social service organizations and has been an investigator on dozens of NIH grants.

MPI: William Cresko, Ph.D., Cresko is a professor of biology and the Executive Director of the Presidential Initiative in Data Science at the University of Oregon. He runs a lab examining the genetic and genomic basis of phenotypic variation in vertebrates. He has extensive experience in the design and analysis of molecular genetics, genetic mapping, Illumina sequencing, and other genomic studies. In addition, Cresko has experience with the development of analytical and numerical biological models, the analysis of large amounts of genomic data, and the development of novel Next Generation Sequencing methodology and computational tools such as RAD-sequencing and the *Stacks* analysis pipeline. He has published over 100 papers that have garnered nearly 16,000 citations. Cresko has been PI numerous NSF grants and several NIH R01 grants from NIGMS and NIEHS. He has also been PI on (a) two large R24 resource grants from ORIP specifically for the development and dissemination of genomic tools, techniques, and resources and (b) a NIGMS P50 Center of Excellence project to study the health effects of microbiome variation on human health using fish models. He is also collaborating with co-PIs Leve and DeGarmo in the Data Science Core of a NIH P50 Center of Excellence project focusing on mitigating the negative consequences of opioid addiction in mothers.

Research Compliance Services
Approved 11/19/2021 - 9/30/2022

MPI: David DeGarmo, Ph.D., DeGarmo is an Associate Research Professor at the University of Oregon Prevention Science Institute and PI of the UO Data Science Core for the P50 DA048756-01 (Leve) centers of excellence Center on Parents and Opioids. DeGarmo has expertise in prevention science methodology and is former director of the Center for Assessment, Statistics and Evaluation (CASE) at the University of Oregon. He has published more than 40 efficacy and effectiveness evaluations, including work on implementation process and fidelity; mediation and moderation in the context of randomized, controlled trials; and multilevel samples. Relevant to the optimization strategies outlined in the present application DeGarmo teaches adaptive research design in the College of Education and is multiple PI of a sequential multiple armed randomized trial (SMART) funded by the Department of Defense entitled “SMART Optimization of a Parenting Program for Active-Duty Families” (W81XWH-16-1-0407). In addition, he is an investigator on the “Supporting our Staff (SOS-19)”, an individually randomized three-arm comparative effectiveness trial with over 1,000 direct line healthcare workers currently under review by PCORI (20-10782). DeGarmo has a longstanding successful history of science collaboration with this team of investigators.

Co-I: Camille Cioffi, Ph.D., Cioffi is a Research Associate at the Prevention Science Institute at the University of Oregon. She has experience in providing leadership and support to multicomponent and multi-site projects, including her role on the CPO, coordination for Dr. Leve's ECHO cohort award from NIH, and Oregon Suicide Prevention and Response for Youth (OSPREY). On the CPO, she has worked closely with community-based agencies to establish research to practice partnerships and has provided support on the administrative core to improve coordination between the center components which includes service on the data science core and science communication committee. Cioffi has worked extensively with the MPIs for this project engaging in weekly meetings with each of the PIs in her various roles. In addition to her role on the CPO and OSPREY, she is presently assisting Drs. Leve and Cresko on the University of Oregon on the COVID-19 Monitoring and Assessment Program as the project Community Liaison, which has included assistance coordinating the implementation of testing sites in Lane County and Marion County. Along with her publications on public health research and implementation science, her commitment to bridging the gap from research to practice is exemplified in her involvement with the federal Research-to-Policy Collaboration, service to Oregon Health Authority collaborations, and co-instruction of Implementation Science coursework.

Co-I: Elizabeth Budd, Ph.D., M.P.H., Budd is the Evergreen Assistant Professor in Prevention Science. Her public health expertise includes community-based participatory research, behavior change-focused interventions among underserved populations, and identifying factors associated with effective implementation of evidence-based disease prevention interventions. Partnering with the local Boys & Girls Club, she recently conducted a two-year community based participatory research project to inform the cultural adaptations and implementation strategies for a health promotion program for adolescent girls from low socioeconomic households, many of whom identified as Latina. Budd has three first-authored and two second-authored publications on the multi-level contextual factors that influence the implementation of evidence-based chronic disease prevention interventions in Australia, Brazil, China, and the United States. She also surveyed a U.S. national sample of 798 Latinx adults and, based on these data, has one manuscript under review in which she examined the moderating effects of perceived neighborhood walkability on the association between perceived racial discrimination stress and having a chronic health condition. Guided by the CDC’s “Promoting Health Equity: A Resource to Help Communities Address Social Determinants of Health,” Budd coordinated preliminary work for the proposed project involving interviews with community stakeholders at Latinx-serving organizations to gather feedback on outreach and testing protocols. In addition to her scholarly work, she has relevant experience with Latinx cultures, including a bachelor’s degree in Spanish Studies, studied abroad in San Jose, Costa Rica, Seville, Spain, and San Salvador, El Salvador; she completed her public health internship in Curritiba, Brazil.

Research Compliance Services
Approved 11/19/2021 - 9/30/2022

Co-I: Stephanie De Anda, Ph.D., De Anda is an Assistant Professor in the College of Education at the University of Oregon. She is a certified Spanish-English bilingual speech-language pathologist and co-directs the Early Dual Language Development Lab, which serves young Latinx infants and toddlers with and without language delays. The lab has many relationships with local organizations serving the Latinx community who refer families and children to be screened by our team. In turn, the lab provides families with screening results, and provide community outreach activities by leading parent and teacher training on healthy language development in for children learning Spanish and English. De Anda has been awarded several NIH grants to support this work that specifically examine young Latinx children and their families.

Co-I: Ellen McWhirter, Ph.D., McWhirter is the Ann Swindells Professor in Counseling Psychology and director of the Spanish Language Psychological Services and Research (SLPSR) Specialization at the University of Oregon. Her scholarship on Latinx adolescent career development, critical consciousness, and risk and protective factors is particularly salient to her roles on this grant. Her contributions include 59 refereed publications, 13 book chapters, and 2 books as well as 140 refereed or invited presentations. A Fulbright Scholar (Universidad de Chile, Santiago, Chile, 2004), her integration of research with service has been recognized with national and university awards. In conjunction with her scholarship, her roles on the executive board of Center for Latina/o and Latin American Studies (CLLAS) and the University of Oregon (UO) Dreamers Working Group, and more than a decade of evaluation work with the César E. Chávez Leadership Conference, provide a strong network of connections with Oregon Latinx communities.

Co-I: Anne Mauricio, Ph.D., Mauricio is an Associate Research Professor and a Family Intervention Scientist at the University of Oregon. She has 18 years of experience collaborating with Latinx communities to develop, implement, and evaluate culturally competent evidence-based interventions, and she has been PI or Co-I on several grants focused on implementation of evidence-based interventions in real-world practice. She is also a licensed psychologist with experience working with Latinx families and children in community mental health settings and training and supervising clinicians in the delivery of culturally competent interventions.

Co-I: John Seeley, Ph.D., Dr. Seeley will serve as Co-Investigator and lead the Implementation Science working group to oversee the implementation and improvement science methodology employed to address the study aims. Seeley is a Professor of Special Education and Clinical Sciences at the University of Oregon with over 30 years of experience in conducting epidemiologic and behavioral health research and currently serves as the Associate Director for the Center on Human Development within the College of Education. Since 2005, Seeley has focused on translational research using effectiveness-implementation hybrid designs to evaluate the implementation and sustainment of evidence-based practices within community settings. He currently serves as the PI on a multisite Collaborative R01 effectiveness-implementation Type 1 hybrid SMART design (R01MH116050) to examine adaptive treatment strategies for intervening with college students experiencing elevated suicidal ideation through college counseling centers. Seeley has more than 250 peer reviewed publications and has served as the PI on more the 20 NIH and DOE research grants.

Co-I: Hannah Tavalire, Ph.D., Tavalire is a Research Associate in the Prevention Science Institute at the University of Oregon. She currently serves as the Scientific Coordinator for the COVID-19 Monitoring and Assessment Program (COVID-19-MAP), working closely with community partners in Lane and Marion Counties to facilitate testing and collect research samples. Tavalire has also served on working groups and task forces as part of an ECHO cohort award and in the Data Science Core of Leve's P50 Center of Excellence award. She has expertise in infectious disease biology, genomics, and has led several field research teams, including a current SARS-CoV-2 testing team.

Research Compliance Services
Approved 11/19/2021 - 9/30/2022

Co-I: Doug Turnbull, Ph.D., Director, UO Genomics Core Facility, Turnbull is director of the Genomics and Cell Characterization Core facility (GC3F) and has extensive experience performing research in molecular biology, genetics, and genomics, as well as managing a large research core facility. His years of experience will be critical for the success of the laboratory testing. He has been co-leading with Dr. Bassham the establishment of molecular laboratory infrastructure for the UO's COVID-19 Monitoring and Assessment Program. Along with Dr. Bassham, he has been guiding the university's effort in adaptation and improvement of molecular testing protocols for SARS-CoV-2 detection in clinical samples.

Co-I: Susan Bassham, Ph.D., Dr. Bassham is a Senior Research Associate in the Institute of Ecology and Evolution, with expertise in high throughput molecular biology applications in genomics and genetics. She has co-led with Dr. Turnbull the establishment of molecular laboratory infrastructure for the UO's COVID-19 Monitoring and Assessment Program. Along with Turnbull, she has guided the university's effort in adaptation and improvement of molecular testing protocols for SARS-CoV-2 detection in clinical samples.

Co-I: Ariana White, MLS (ASCP), Technical Supervisor, Testing Lab,Ms. White has responsibility for ensuring that the processes of sample acquisition, testing, and reporting are CLIA and HIPAA compliant in order to guarantee high-quality, confidential diagnostic work on behalf of the patients participating in this effort. Additionally, she will ensure compliance with State of Oregon Health Department reporting standards. She has 24 years of experience as a clinical diagnostician in multiple healthcare environments.

Co-I: Jeff Gau, M.S., Mr. Gau is a Research Associate in the College of Education at the University of Oregon. He has provided data management and analytic support on over 50 Dept. of Education and NIH grants and had over 90 peer reviewed publications.

Co-I: Jake Searcy, Ph.D., Searcy currently leads the Data Science efforts of UO COVID MAP project, which consists of maintaining sample data pipelines and modeling of covid19 spread. Searcy will serve the critical role of ensuring that the project can utilize and build upon the data processes developed for the UO MAP project. Searcy is the Associate Director of AI in UO's Research Advanced Computing Research Services, and UO's Presidential Data Science Initiative. In this role, Searcy works with researchers across UO to successfully utilize new AI techniques in their endeavors. He has extensive knowledge of statistical analysis and data systems from his time as an Analyst at Ford Motor Company and his background as a particle physicist working on the ATLAS experiment at CERN.

Co-I: Emily Beck, Ph.D., Beck is a Research Associate and Statistical Analyst in Data Science Research Services as part of the Presidential Data Science Initiative at the University of Oregon. She is currently a member of the data science team and the campus modeling projects for the UO's COVID-19 MAP activities. She has a background in genomics, bioinformatics, and statistics focusing on systems biology and complex molecular and genetic interactions underlying microbial interfaces with host immunity.

2. Roles and Research Duties

Principal Investigators will assume responsibility for all scientific, administrative, financial, and operational aspects of the project.

Co-Investigators will:

- assist the PIs with administrative functioning of the overall project
- assist in facilitating the community-based participatory approach

Research Compliance Services
Approved 11/19/2021 - 9/30/2022

- support the project’s collaboration with key community stakeholders and interface with county collaboratives
- interface with funded RADx-UP Social, Ethical and Behavioral Implications program grantees (SEBI, NOT-OD-20-119) and other RADx-UP field sites to support novel research on social, ethical and behavioral implications of testing in underserved and/or vulnerable populations
- contribute to activities to support implementation of the proposed interventions
- oversee the implementation and improvement science methodology employed to address the study aims
- assist in administrative and communication aspects of the testing project between working groups and help coordinate with the CDCC
- coordinate testing activities across sites and supervise field research staff
- serve as expert technical consultant for molecular testing pipelines, including aiding in continuing improvement in testing throughput and in detection sensitivity for SARS-CoV-2 in biological samples
- assume responsibility for ensuring that the processes of sample acquisition, testing, and reporting are CLIA and HIPAA compliant
- serve as data analytic support
- serve as a consultant on data processes and data modeling
- play a leadership role in the design, optimization, and implementation of data science tools and approaches.

Research Assistants will support the implementation of testing sites throughout Oregon and will provide implementation support to County collaborators. RAs will also collect individual and community level data on intervention effectiveness and assist with data analyses and dissemination activities.

3. Training and Oversight

Co-investigators and research assistants will perform research activities within the scope of their training.

Training for research assistants who collect data from participants will be provided by the project coordinator. Training will include how to sanitize devices used for data collection, relevant human subjects training, and standardized processes and procedures for data collection.

Promotores will provide services similar to what they would provide as usual for community-based organization. Given they are part of the research study, but will not be responsible for answering questions about the research or collecting informed consent or assent, and given the possible limited understanding of academic processes and procedures, we are proposing an alternative human subjects training plan for these individuals. Specifically, a researcher with human subjects training will provide human subjects training during the promotores training already being conducted by the research team. All promotores will be added to the research plan and will complete Individual Investigator Agreements (IIA)s if they are not affiliated with the UO. Alternative training procedure and training dates will be documented in the research records.

4. Translator

At least two people who are proficient in Spanish will review all written study materials before providing materials to participating county health departments and community-based organizations.

Research Compliance Services
Approved 11/19/2021 - 9/30/2022
