# Supplementary material for: Effectiveness of a COVID-19 Testing Outreach Intervention for Latinx Communities: A Cluster Randomized Trial
Source: JAMA Netw Open. 2022 Jun 16;5(6):e2216796. doi: 10.1001/jamanetworkopen.2022.16796 (PMC9204550; doi:10.1001/jamanetworkopen.2022.16796)
Supplement: Supplement 2. — Data Sharing Statement [file jamanetwopen-e2216796-s00.pdf]

Data Sharing Statement

DeGarmo. Effectiveness of a COVID-19 Testing Outreach Intervention for Latinx Communities. *JAMA Netw Open*. Published June 16, 2022. doi:10.1001/jamanetworkopen.2022.16796

Data
Data available: Yes
Data types: Deidentified participant data, Data dictionary
How to access data: https://radx-up.org/learning-resources/cdes/
When available: With publication

Supporting Documents
Document types: Statistical/analytic code, Informed consent form
How to access documents: degarmo@uoregon.edu
When available: With publication

Additional Information
Who can access the data: researchers whose proposed use of the data has been approved
Types of analyses: for any purpose
Mechanisms of data availability: with signed data access agreement
